# Comparison of ^68^Ga-PSMA PET and mpMRI for prostate cancer local staging: a comprehensive review and direct meta-analysis

**DOI:** 10.3389/fonc.2024.1410229

**Published:** 2024-11-01

**Authors:** Xinyu Jin, Yijie Cai, Xiaolu Ren

**Affiliations:** ^1^ Department of Paediatrics, Shanxi Medical University, Taiyuan, China; ^2^ Department of Second Clinical Medical College, Shanxi Medical University, Taiyuan, China; ^3^ Department of Radiology, General Hospital of Ningxia Medical University, Yinchuan, Ningxia, China

**Keywords:** ^68^Ga-PSMA PET, mpMRI, local staging, prostate cancer, meta-analysis

## Abstract

**Purpose:**

This meta-analysis is conducted to evaluate the comparative diagnostic efficacy of ^68^Ga-PSMA PET vs. mpMRI in detecting local staging of prostate cancer(PCa).

**Methods:**

A comprehensive search was conducted in the PubMed and Embase databases to identify publications up to February 2024. The analysis included studies that evaluated the direct comparison of ^68^Ga-PSMA PET and mpMRI for local staging of prostate cancer. The reliability of the analyzed studies was evaluated using the QUADAS-2 tool.

**Results:**

The meta-analysis included 10 articles involving 505 patients, which revealed that both ^68^Ga-PSMA PET and mpMRI had similar sensitivities and specificities in detecting extracapsular extension(ECE) and seminal vesicle invasion(SVI). The sensitivities for ECE were 0.56 (95% CI: 0.41-0.71) for ^68^Ga-PSMA PET and 0.57 (95% CI: 0.43-0.71) for mpMRI, and specificities were both 0.84 (^68^Ga-PSMA PET 95% CI: 0.75-0.91, mpMRI 95% CI: 0.76-0.91).For SVI, sensitivities were 0.57 (95% CI: 0.46-0.68) for ^68^Ga-PSMA PET and 0.70 (95% CI: 0.60-0.80) for mpMRI, with specificities of 0.92 (95% CI: 0.86-0.96) for ^68^Ga-PSMA PET and 0.94 (95% CI: 0.89-0.98) for mpMRI. There were no notable variations in sensitivity or specificity between the two methods for detecting ECE and SVI (*P* = 0.89 and 0.93 for ECE, 0.09 and 0.57 for SVI).

**Conclusions:**

This meta-analysis indicates that ^68^Ga-PSMA PET has similar sensitivity and specificity to mpMRI in local prostate cancer staging. Nevertheless, the limited study sample size calls for further, larger prospective studies to validate these findings.

**Systematic review registration:**

https://www.crd.york.ac.uk/PROSPERO/display_record.php?RecordID=522438, identifier CRD42024522438.

## 1 Introduction

Prostate cancer is a major public health issue, being among the most prevalent cancers in males globally (1).Around 30% of people diagnosed with prostate cancer undergo curative treatment, yet between 20-50% encounter biochemical recurrence within ten years (2). Timely diagnosis is crucial for improving survival rates by extending life expectancy, especially in detecting extracapsular extension and seminal vesicle invasion (3).

Traditional diagnostic tools for PCa, including computed tomography (CT), ultrasound, and histopathological biopsy, have been the mainstay in clinical practice (4, 5). However, these modalities present limitations in sensitivity and specificity, particularly in detecting extracapsular extension and seminal vesicle invasion (6). CT and ultrasonography demonstrate constrained resolution capabilities when identifying minuscule anatomical structures and discerning subtle tissue contrasts, frequently resulting in the oversight of incipient stages of ECE and SVI in prostate cancer (7). Conversely, while pathology biopsies provide a more direct approach, they may overlook areas of ECE/SVI that are not included in the sample and are susceptible to errors inherent in the sampling process (8). Their efficacy in accurately staging PCa, especially in advanced cases, is often constrained, leading to potential underestimation of the disease (4).

Prostate-Specific Membrane Antigen(PSMA), a protein found in abundance on the exterior of prostate cancer cells, is a crucial focus for both diagnosing and treating prostate cancer (9, 10). PET scans that target PSMA, particularly those utilizing the ^68^Ga-labeled PSMA ligand, are frequently used in prostate cancer imaging (11). ^68^Ga-PSMA PET is known for its high sensitivity in detecting PCa cells, whereas mpMRI excels in detailed soft-tissue characterization (12, 13). Despite their individual strengths, a comparative analysis of their effectiveness in the local staging of PCa, especially in discerning ECE and SVI, is not well-established due to a lack of comprehensive head-to-head studies (14).

To fill this knowledge void, a systematic review and meta-analysis will be conducted to compare the diagnostic precision of ^68^Ga-PSMA PET and mpMRI for local staging of PCa. The goal is to assess their effectiveness in identifying ECE and SVI, offering a detailed insight into their contributions to PCa treatment and assisting healthcare providers in selecting the optimal diagnostic approach.

## 2 Methods

The meta-analysis adhered to the PRISMA-DTA guidelines for reporting systematic reviews and meta-analyses of diagnostic test accuracy (15). The protocol for this meta-analysis has been registered with PROSPERO under registration number CRD42024522438.

### 2.1 Search strategy

A thorough investigation was carried out in the PubMed and Embase repositories to locate existing publications until February 2024.The search utilized the keywords ‘^68^Ga-PSMA PET’, ‘mpMRI’, ‘PSMA’, and ‘Prostate Cancer’. Additional information can be found in **Supplementary Table 1**. The identification of relevant articles was done through manual searches of the reference lists in the included studies.

### 2.2 Inclusion and exclusion criteria

For studies to be eligible for inclusion in this meta-analysis, they needed to evaluate the diagnostic accuracy of ^68^Ga-PSMA PET and mpMRI in determining the local staging of prostate cancer. The included studies had to meet specific criteria: (1) population: patients with prostate cancer undergoing local staging; (2) interventions: use of ^68^Ga-PSMA PET; (3) comparators: use of mpMRI; (4) outcomes: sensitivity and specificity; and (5) study type: retrospective studies and prospective studies.

Articles that were duplicates, lacked full texts, consisted of editorials, letters, case reports, reviews, meta-analyses, items with irrelevant titles or abstracts, and publications in languages other than English were excluded. Additionally, studies without enough data to calculate sensitivity or specificity of the imaging technique being studied were also excluded.

### 2.3 Quality assessment

Two researchers assessed the studies’ quality using the QUADAS-2 tool (16, 17), which evaluates diagnostic performance in four main areas: patient selection, index test, reference standard, and flow and timing. Each study was categorized as having high, low, or unclear bias risk.

### 2.4 Data extraction

Data extraction for all included papers was carried out independently by two researchers. The data that were extracted included: (1) the author; (2) year of publication; (3) study characteristics including country, study design, analysis, outcome; (4) patient characteristics including number of patients, PSA level, mean/median age, Gleason score, reference standard; (5) technical characteristics including types of imaging tests, scanner modality for PET, scanner modality for mpMRI, radiotracer dose, image analysis, image analysis, TP, FP, FN, TN for ECE(PET), TP, FP, FN, TN for ECE(mpMRI), TP, FP, FN, TN for SVI(PET), TP, FP, FN, TN for SVI (mpMRI).

### 2.5 Statistical analysis

The DerSimonian and Laird method was utilized for random-effects meta-analysis to evaluate sensitivities and specificities, accounting for variability between studies. Sensitivities and specificities were subsequently converted using the Freeman-Tukey double inverse sine transformation to stabilize the variance of proportion data and improve the reliability of pooled estimates (18). Confidence intervals were determined using the Jackson technique. Heterogeneity within and across groups was assessed utilizing the Cochrane Q and I² statistics. If significant diversity was observed among the studies (*P*<0.10 or I^2^ > 50%), a sensitivity analysis was performed to determine the reasons for the discrepancies.

Funnel plots and Egger’s test were used to evaluate publication bias. Statistical tests had to reach a significance level of *P* < 0.05. Statistical analysis and graphical representation were conducted using R software version 4.3.2.

## 3 Results

### 3.1 Study selection

**Figure 1:**
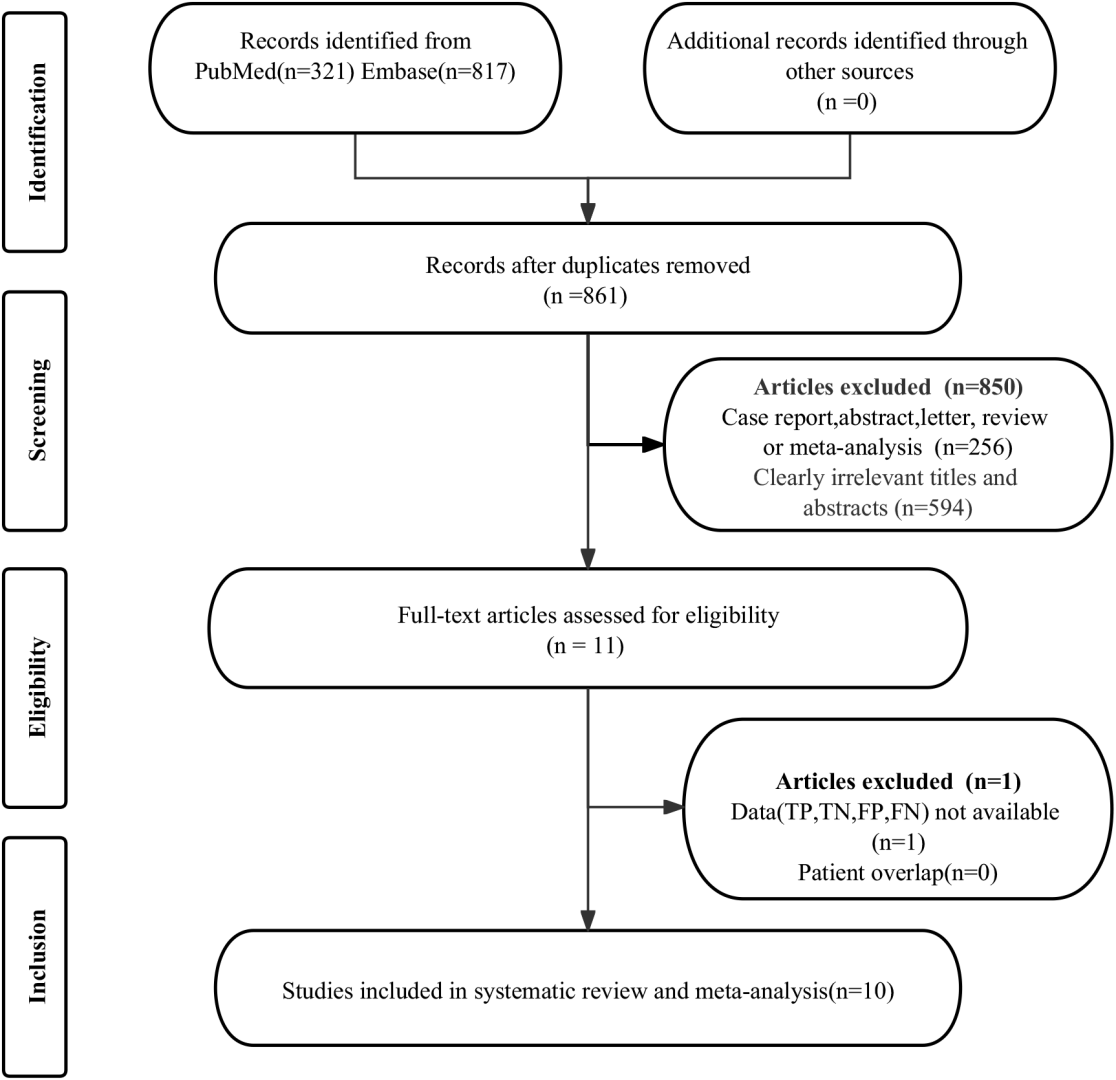
The flow diagram of study selection.

The initial search found a total of 1138 published works. However, 277 studies were duplicates, and 850 were considered ineligible and removed from further review. After evaluating the full manuscripts of the remaining 11 studies, one was disqualified for missing critical data (TP, FP, FN, and TN). Ultimately, the meta-analysis included 10 studies (19–28) that evaluated the diagnostic efficacy of ^68^Ga-PSMA PET and mpMRI. The article selection process, as detailed in the PRISMA flowchart (29), is depicted in **Figure 1**.

### 3.2 Assessment of study description and quality

The 10 qualifying studies included 505 patients diagnosed with prostate cancer, aged between 24 and 81. Out of these studies, 8 were retrospective (19, 21, 22, 24–28), while 2 were prospective (20, 23). All 10 studies used patient-based analyses, consistently referring to pathology as the standard for analysis. 8 studies (21–27) provided data on the sensitivity and specificity of two diagnostic tools for extracapsular extension. Furthermore, 8 studies included statistics on the sensitivity and specificity for seminal vesicle invasion (19–21, 23–26, 28). The details of the study and methodology for ^68^Ga-PSMA PET and mpMRI were outlined in **Tables 1**, **2**.

**Table 1:** Study and patient characteristics of the included studies.

Author	Year	Study characteristics				Number of patients (lesion)	Patient characteristics			
		Country	Study design	Analysis	Outcome		PSA level (ng/ml)	Age(year)	Gleason Score	Reference standard
Ucar et al. (19)	2022	Turkey	Retro	PB	ECE,SVI	49	mean:21.11	mean:66.18	NA	Pathology
Tayara et al. (20)	2023	Poland	Pro	PB	ECE,SVI	74	median:13	median:66	NA	Pathology
Stasiak et al. (21)	2023	Brazil	Retro	PB	ECE,SVI	65	median:6.8	mean:69.3	Gleason ≤ 6(12.5%) Gleason=7(71.9%) Gleason≥8 (15.6%)	Pathology
Arslan et al.	2020	Turkey	Retro	PB	ECE	39	median:9.53	median:62.47	Gleason=7 (84.6%) Gleason≥8 (15.4%)	Pathology
Çelen et al. (23)	2020	Turkey	Pro	PB	ECE,SVI	30	mean:9.49	mean:65.07	Gleason ≤ 6(23.3%) Gleason=7(40%) Gleason≥8 (36.7%)	Pathology
Yilmaz et al. (24)	2019	Turkey	Retro	PB	ECE,SVI	24	mean:12.0	mean:62.8	Gleason ≤ 6(12.5%) Gleason=7(66.6%) Gleason≥8 (20.9%)	Pathology
Chen et al. (25)	2020	China	Retro	PB	ECE,SVI	54	median:13.30	median:69	Gleason ≤ 6(11.1%) Gleason=7(53.7%) Gleason≥8 (35.2%)	Pathology
Koseoglu et al.	2020	Turkey	Retro	PB	ECE,SVI	81	median:7	median:65	NA	Pathology
Muehlematter et al. (26)	2019	Switzerland	Retro	PB	ECE,SVI	40	median:8.12	mean:63	Gleason=7(20%) Gleason≥8 (80%)	Pathology
Skawran et al. (27)	2022	Switzerland	Retro	PB	ECE	49	median:18.3	median:66	Gleason=7 (26.5%) Gleason≥8 (73.5%)	Pathology

Pro, prospective; Retro, retrospective; PB, patient-based; NA, not available; ECE, extracapsular extension; SVI, seminal vesicle infiltration.

**Table 2:** Technical aspects of included studies.

Author	Year	Technical characteristics								
		Types of imaging tests	Scanner Modality for PET	Scanner Modality for mpMRI	Radiotracer dose	Image analysis	TP,FP,FN,TN for ECE (PET)	TP,FP,FN,TN for ECE (mpMRI)	TP,FP,FN,TN for SVI(PET)	TP,FP,FN,TN for SVI (mpMRI)
Ucar et al. (19)	2022	68Ga- PSMA PET/CT vs. mpMRI	PMT‐based TOF PET/CT with 64‐slice CT(Discovery710;GE Healthcare) And four‐ring PMT‐based BGO PET/CT with16‐slice CT (Discovery IQ; GE Healthcare)	1.5 T GE Optima MR450w (General Electric) system	189.5MBq	Visual and semiquantitative		TP:18,FP:1,FN:9,TN:12	TP:9,FP:4,FN:5,TN:22	TP:10,FP:6,FN:4,TN:20
Tayara et al. (20)	2023	68Ga- PSMA PET/CT vs. mpMRI	Biograph 64 True Point scanner(Siemens Medical Solutions Inc.,Malvern,PA,USA)	1.5T and 3.0T (Siemens (Berlin,Gremany),PhilipsHealthcare (Amsterdam,The Netherlands),and General Electric (Boston,MA,USA))	2MBq/kg	Visual		TP:20,FP:4,FN:32,TN:18	TP:8,FP:6,FN:18,TN:42	TP:17,FP:5,FN:9,TN:43
Stasiak et al. (21)	2023	68Ga- PSMA PET/CT vs. mpMRI	NA	1.5-T or 3.0-T scanners	1.8–2.2 MBq	Visual and semiquantitative	TP:1,FP:5,FN:6,TN:53	TP:2,FP:17,FN:5,TN:41	TP:9,FP:0,FN:6,TN:50	TP:12,FP:1,FN:3,TN:49
Arslan et al	2020	68Ga- PSMA PET/CT vs. mpMRI	GE Discovery 710 (General Electric, Milwaukee WI),GE Discovery IQ(General Electric, Milwaukee WI),or Siemens (Siemens,Erlangen,Germany) Biograph 20 mCT	3.0-T MR scanner(Siemens Healthineers,Magnetom Skyra,Erlangen,Germany)	NA	Visual	TP:10,FP:9,FN:6,TN:14	TP:9,FP:4,FN:7,TN:19	NA	NA
Çelen et al. (23)	2020	68Ga- PSMA PET/CT vs. mpMRI	PET-CT unit (Gemini TF TOF PET-CT; Philips, Cleve gland, OH, USA)	1.5-T superconducting magnet (Ingenia, Philips Medical Systems, The Netherlands)	185Mbq	Visual and semiquantitative	TP:9,FP:6,FN:8,TN:7	TP:13,FP:5,FN:4,TN:8	TP:5,FP:5,FN:1,TN:19	TP:5,FP:3,FN:1,TN:21
Yilmaz et al. (24)	2019	68Ga- PSMA PET/CT vs. mpMRI	ITG semi‐automated generator(Munich,Germany)	3.0‐T MR unit(Verio;Siemens Medical Solutions,Erlangen, Germany)	175MBq	Visual	TP:3,FP:1,FN:7,TN:13	TP:9,FP:2,FN:1,TN:12	TP:3,FP:2,FN:1,TN:18	TP:4,FP:1,FN:0,TN:19
Chen et al. (25)	2020	68Ga-PSMA PET/MRI vs. mpMRI	uMI 780 PET-CT scanner [United Imaging Healthcare (UIH), Shanghai, China]	3.0-T MR scanner (Achieva 3.0 T TX, Philips Medical Systems, The Netherlands)	135.72 MBq	Visual and semiquantitative	TP:31,FP:2,FN:6,TN:15	TP:20,FP:1,FN:17,TN:16	TP:9,FP:5,FN:3,TN:37	TP:8,FP:3,FN:4,TN:39
Koseoglu et al.	2020	68Ga-PSMA PET/MRI vs. mpMRI	NA	3-T(MagnetomSkyra;Sie mensAG)	NA	Visual and semiquantitative	TP:8,FP:7,FN:22,TN:44	TP:15,FP:4,FN:15,TN:47	TP:3,FP:3,FN:8,TN:67	TP:6,FP:1,FN:5,TN:69
Muehlematter et al. (26)	2019	68Ga-PSMA PET/MRI vs. mpMRI	3.0-T hybrid scanner (SIGNA PET/MR; GE Healthcare, Waukesha, Wis)	1.5-T or a 3.0-T whole-body MRI system	131 MBq	Visual	TP:6,FP:3,FN:6,TN:25	TP:3,FP:2,FN:9,TN:26	TP:3,FP:2,FN:2,TN:33	TP:2,FP:1,FN:3,TN:34
Skawran et al. (27)	2022	68Ga-PSMA PET/MRI vs. mpMRI	3.0 T hybrid scanner (SIGNA PET/MR; GE Healthcare, Waukesha, USA)	3.0 T clinical MRI scanners (MAGNETOM Skyra, Siemens Healthineers, Erlangen, Germany)	134 MBq	Visual	TP:7,FP:5,FN:6,TN:31	TP:9,FP:7,FN:4,TN:29	NA	NA

TP, true positive; TN, true negative; FP, false positive; FN, false positive; NA, not available.

**Figure 2:**
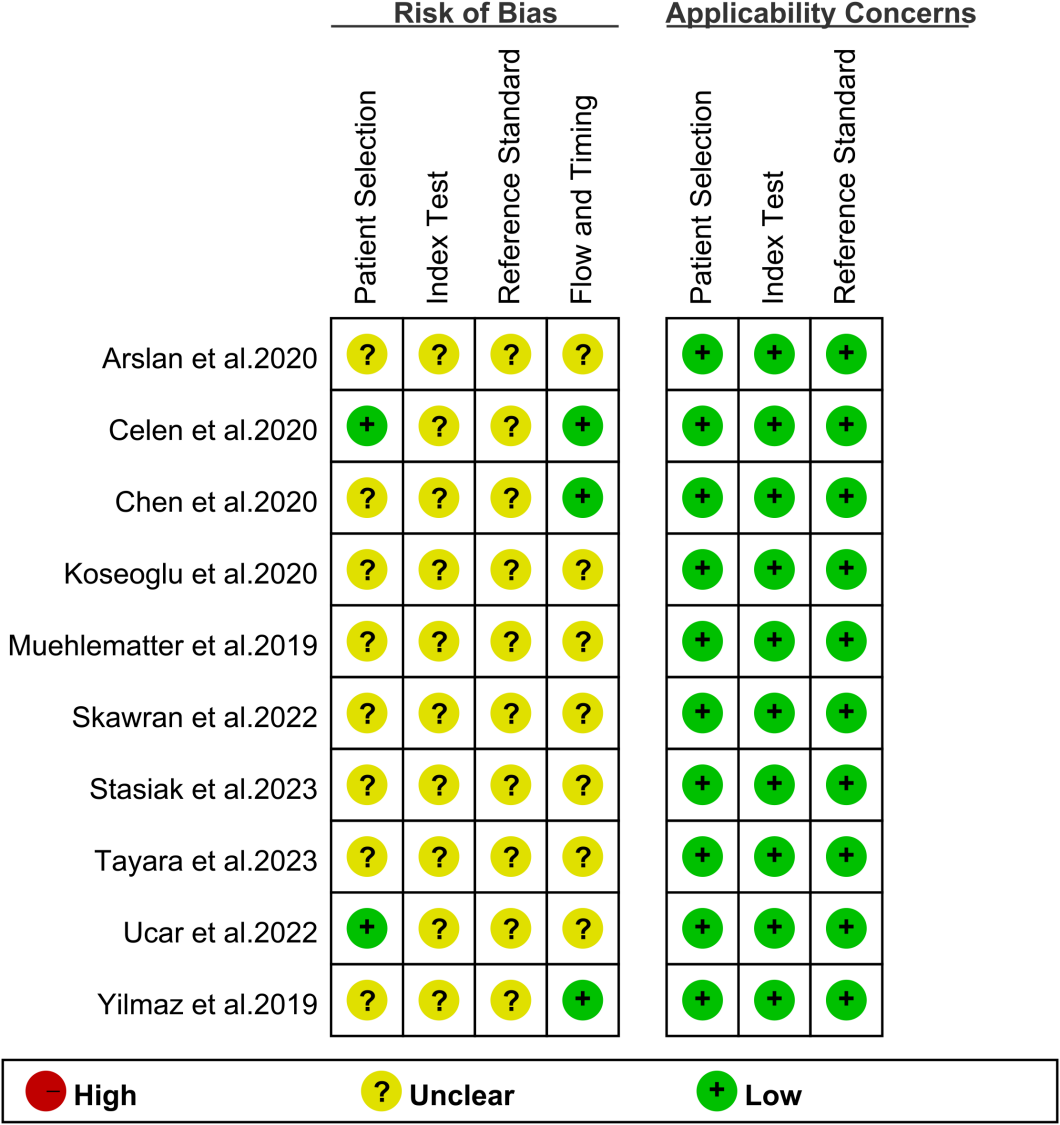
Evaluation of study reliability and relevance using QUADAS-2 for diagnostic accuracy assessments.

**Figure 2** displays the potential bias in each study as determined by the QUADAS-2 tool. Eight studies were deemed ‘unclear’ in terms of patient selection due to a lack of information on the inclusion of consecutive patients, indicating potential selection bias. All 10 studies received an ‘unclear’ rating for the index test because visual grading thresholds could not be identified, raising concerns about detection bias. Regarding the reference standard, all 10 studies were also rated as ‘unclear’ due to uncertainty about whether the final diagnosis was independently made by two or more doctors, which may impact the reliability of the reference standard. Seven studies were classified as ‘unclear’ in terms of flow and timing criteria due to uncertainty about the correct timing between the diagnostic test and the gold standard evaluation, which could introduce variability in diagnostic accuracy. Overall, while these potential biases may have some impact, the quality of the included literature is generally acceptable.

### 3.3 Comparing the sensitivity of ^68^Ga-PSMA PET and mpMRI in identifying extracapsular extension in prostate cancer

**Figure 3:**
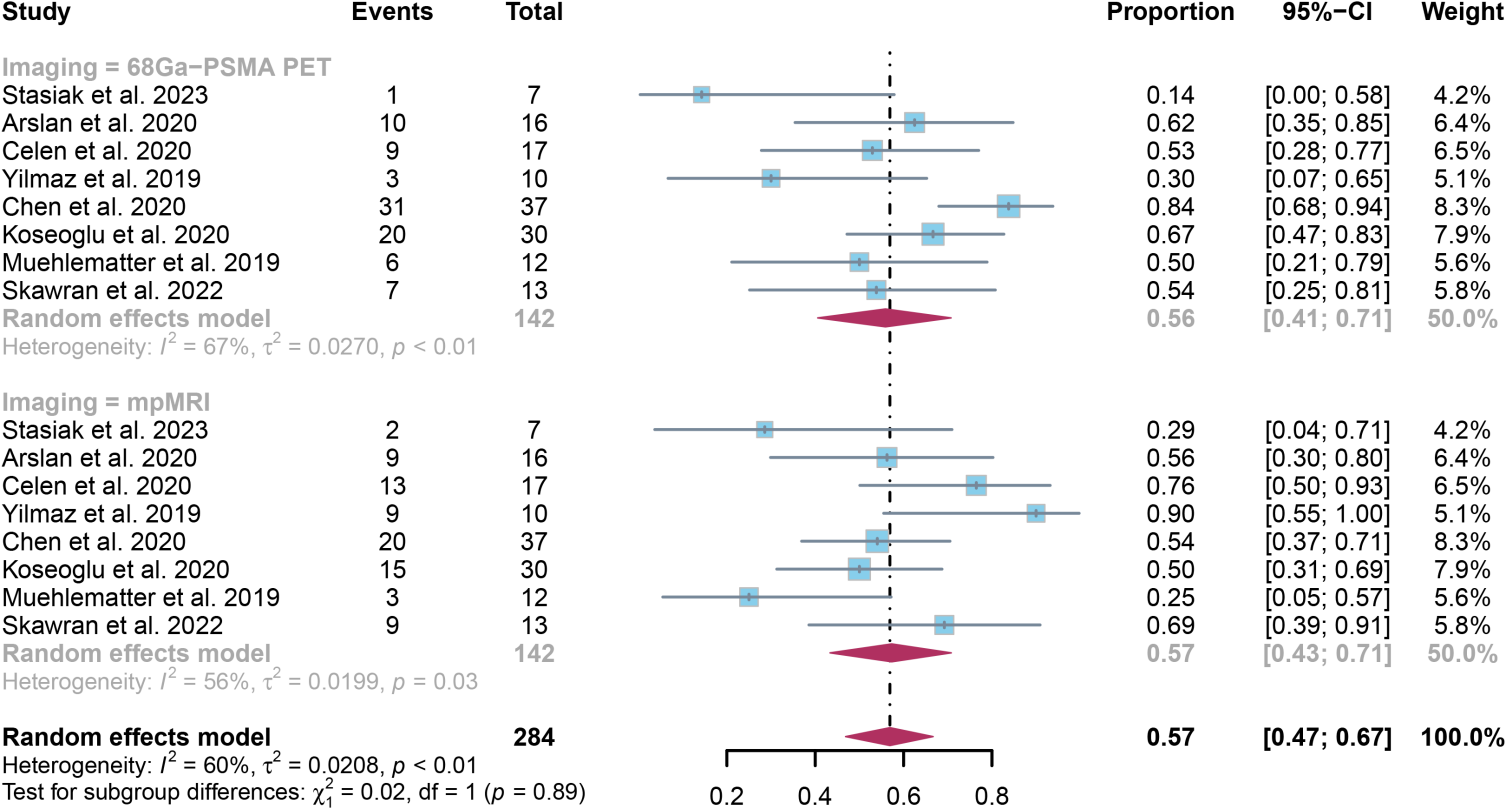
Forest plot showing the pooled sensitivities of ^68^Ga-PSMA PET and mpMRI in extracapsular extension of prostate cancer patients.

The evaluation comprised 8 research studies, with a sensitivity of 0.56 (95% CI:0.41-0.71) in identifying ECE of prostate cancer through ^68^Ga-PSMA PET, whereas mpMRI demonstrated an overall sensitivity of 0.57 (95% CI:0.43-0.71) (**Figure 3**). There was no notable significant difference in sensitivity between ^68^Ga-PSMA PET and mpMRI (*P* = 0.89) (**Figure 3**).

The overall sensitivity of ^68^Ga-PSMA PET and mpMRI demonstrated I^2^ percentages of 67% and 56% correspondingly. Upon leave-one-out sensitivity analysis, the I^2^ percentage for ^68^Ga-PSMA PET dropped to 33% when Chen’s study was omitted, indicating it may have caused heterogeneity. Likewise, excluding Yilmaz’s or Muehlematter’s study led to reduced I^2^ percentages for mpMRI, reaching 42% and 44% respectively, suggesting each study could have contributed to heterogeneity (**Supplementary Figures 1, 2**).

### 3.4 Comparing the specificity of ^68^Ga-PSMA PET and mpMRI in detecting extracapsular extension of prostate cancer

**Figure 4:**
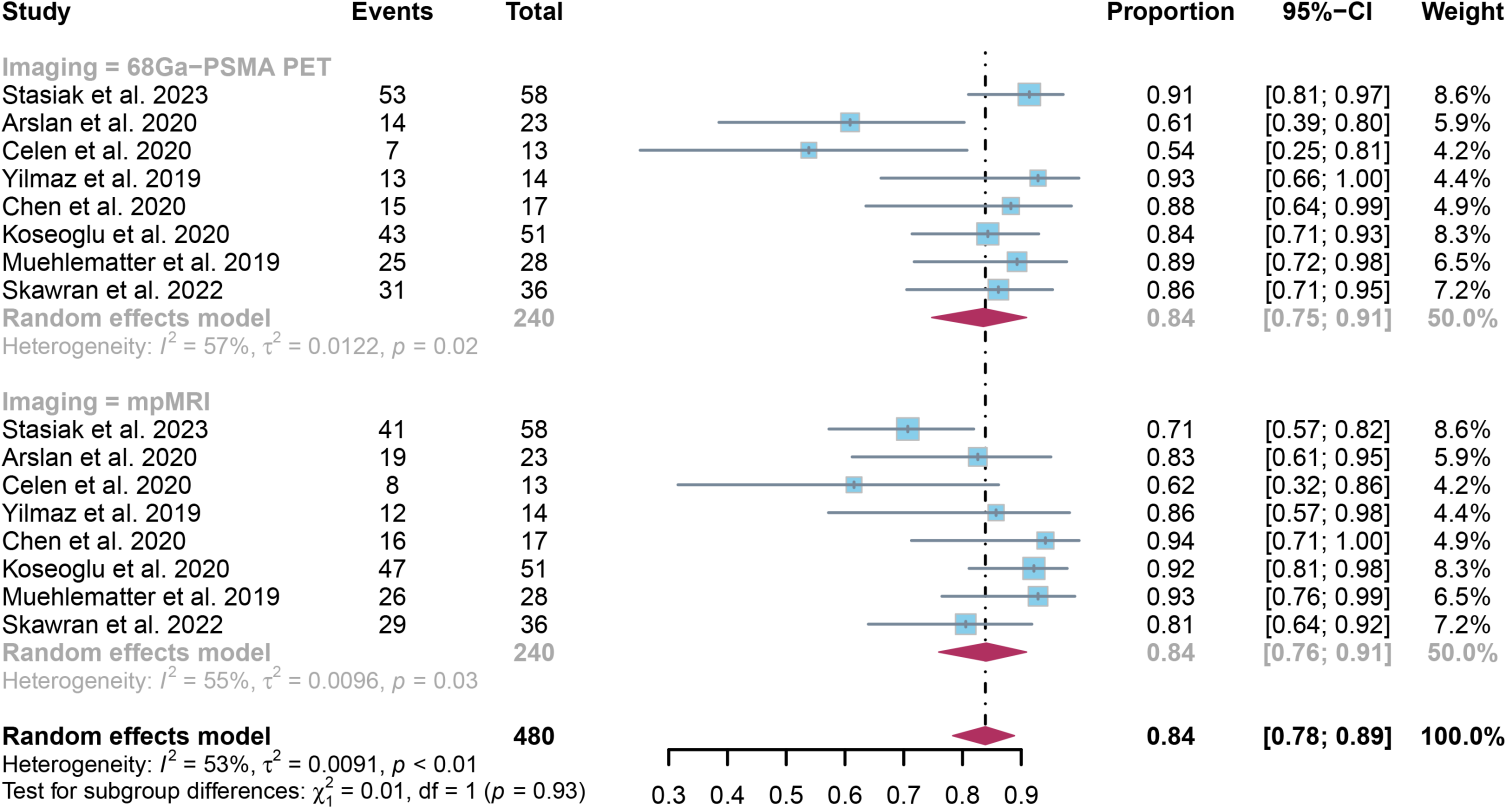
Forest plot showing the pooled specificities of ^68^Ga-PSMA PET and mpMRI in extracapsular extension of prostate cancer patients.

The examination revealed that the specificity was 0.84 (95% CI:0.75-0.91), with mpMRI demonstrating a comparable specificity of 0.84 (95% CI:0.76-0.91). There was no notable difference in overall specificity between ^68^Ga-PSMA PET and mpMRI (*P* = 0.93) (**Figure 4**).

The specificity of PET and mpMRI demonstrated I² percentages of 57% and 55%, respectively. Upon further leave-one-out sensitivity analysis, it was found that excluding either Arslan’s or Celen’s research decreased the I² value for ^68^Ga-PSMA PET to 34% and 40%, respectively, indicating they may be sources of heterogeneity. Similarly, the removal of Stasiak’s or Koseoglu’s study led to a reduction in I² values for mpMRI to 34% and 45%, respectively, highlighting their potential impact on heterogeneity. (**Supplementary Figures 3, 4**).

### 3.5 Comparing the sensitivity of ^68^Ga-PSMA PET and mpMRI in identifying seminal vesicle invasion in prostate cancer

**Figure 5:**
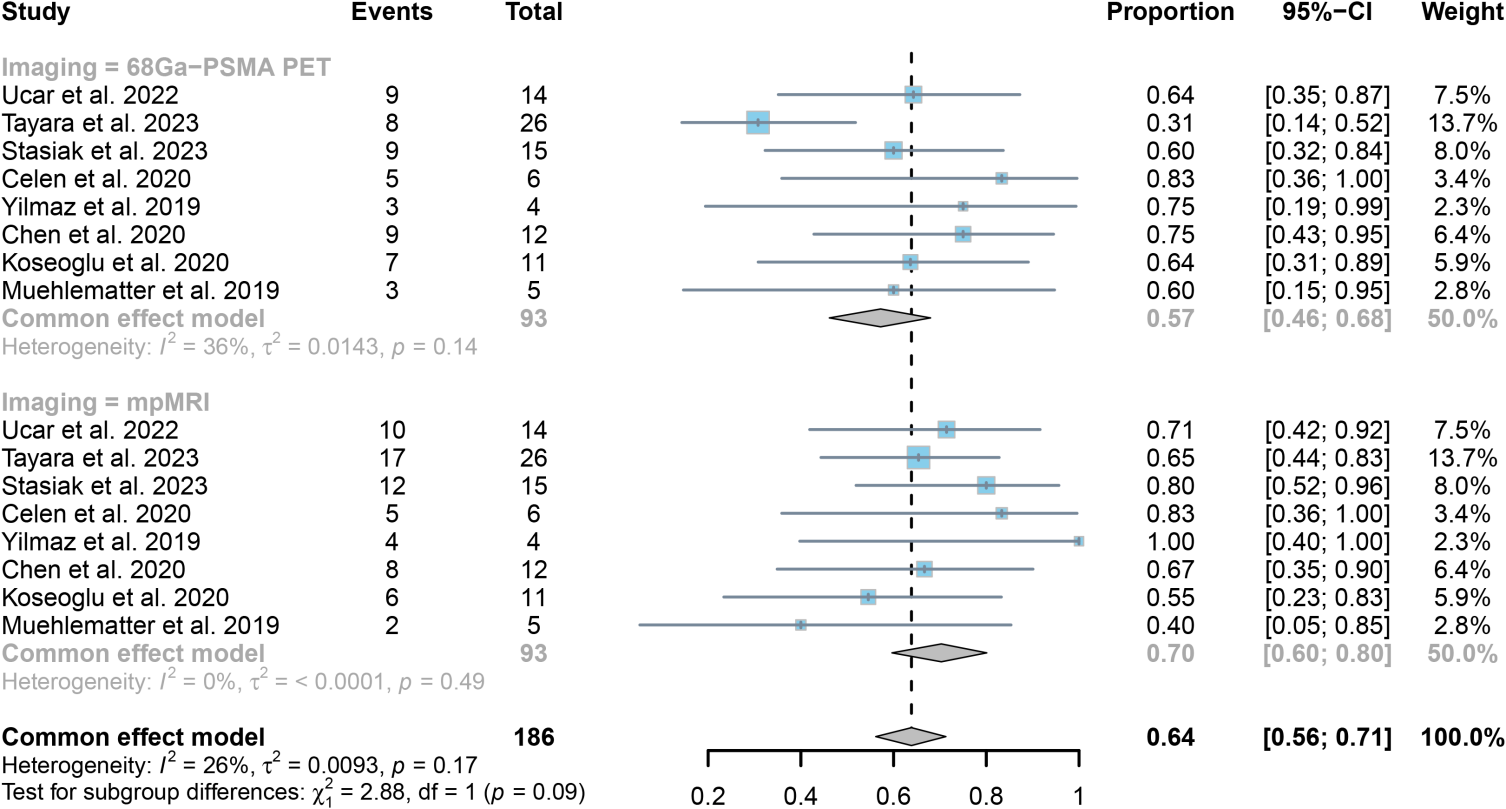
Forest plot showing the pooled sensitivities of ^68^Ga-PSMA PET and mpMRI in seminal vesicle invasion of prostate cancer patients.

The analysis of 8 research studies revealed that ^68^Ga-PSMA PET had a sensitivity of 0.57 (95% CI:0.46-0.68) for detecting SVI in prostate cancer, while mpMRI had a sensitivity of 0.70 (95% CI:0.60-0.80), as shown in **Figure 5**. There was no significant difference in sensitivity between the two imaging methods (*P* = 0.09) (**Figure 5**).

The collective responsiveness of PET and mpMRI indicated I^2^ values of 36% and 0%, respectively, indicating that both techniques exhibit acceptable heterogeneity in identifying SVI.

### 3.6 Comparing the specificity of ^68^Ga-PSMA PET and mpMRI in detecting seminal vesicle invasion in prostate cancer patients

**Figure 6:**
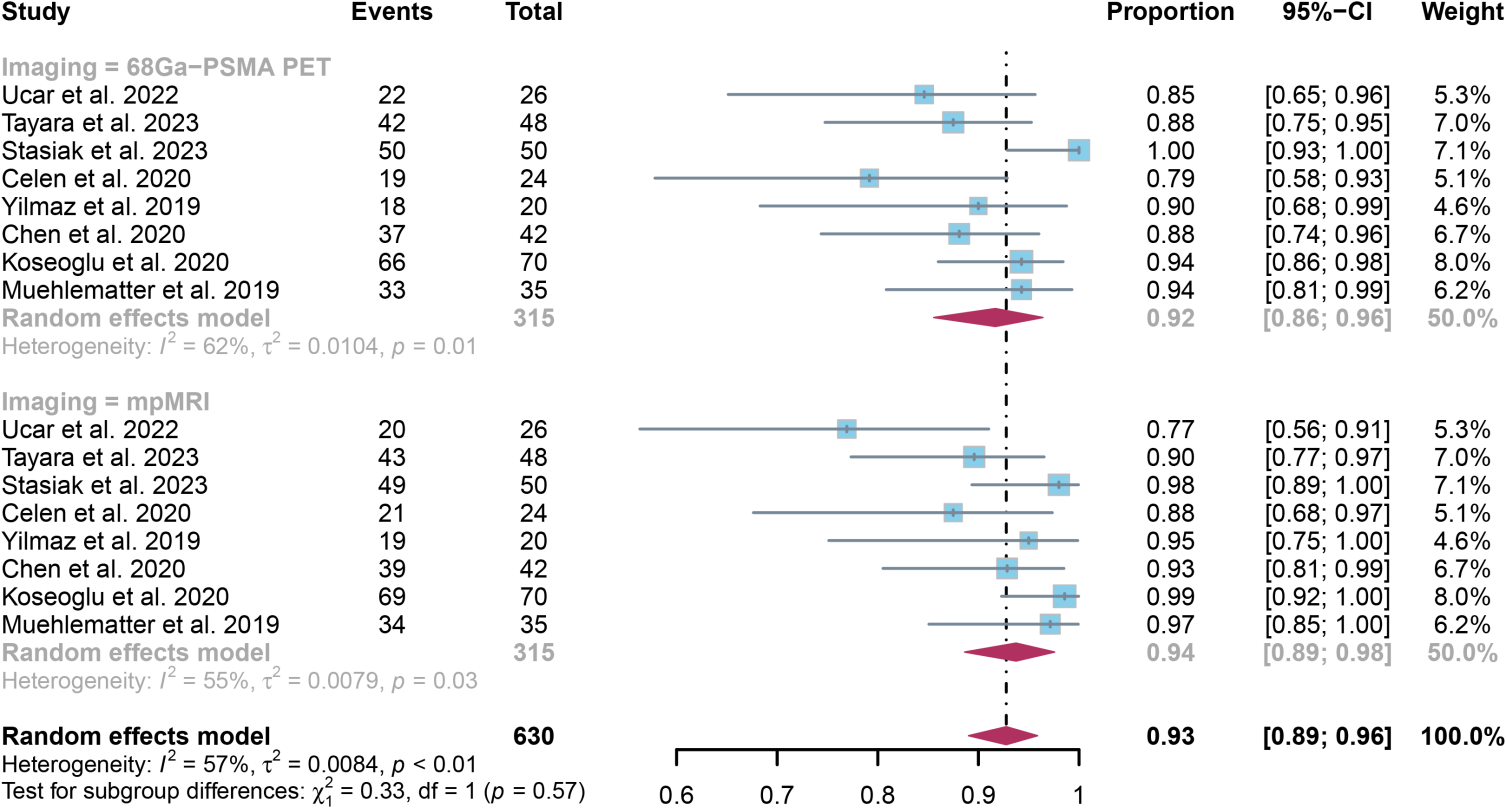
Forest plot showing the pooled specificities of ^68^Ga-PSMA PET and mpMRI in seminal vesicle invasion of prostate cancer patients.

Analysis of 8 studies with 417 patients found that ^68^Ga-PSMA PET had an specificity of 0.92 (95% CI:0.86-0.96) in detecting SVI in prostate cancer, while mpMRI had an specificity of 0.94 (95% CI:0.89-0.98), as illustrated in **Figure 6**. The methods did not show any significant difference in specificity, with a *P* value of 0.57.

The overall specificity of ^68^Ga-PSMA PET and mpMRI showed I² values of 62% and 55%, in that order. Upon leave-one-out sensitivity analysis, the I^2^ value for ^68^Ga-PSMA PET dropped to 0% when excluding Stasiak’s study, indicating it may be a significant factor contributing to heterogeneity. For mpMRI, after omitting Ucar’s study or Koseoglu’s study, the I^2^ value reduced to 27% and 44%, respectively.(**Supplementary Figures 5, 6**)

### 3.7 Examining the sensitivity and specificity of various ^68^Ga-PSMA PET imaging techniques (PET/CT and PET/MRI) and mpMRI in identifying extracapsular extension and seminal vesicle invasion in cases of prostate cancer

The information in **Table 3** outlines the effectiveness of ^68^Ga-PSMA PET/CT and mpMRI in detecting ECE in prostate cancer, with a collective sensitivity of 0.43 (95% CI:0.24-0.64) and 0.66 (95% CI:0.41-0.87) respectively. There was no significant difference in sensitivity between these methods (*P* = 0.17). Additionally, ^68^Ga-PSMA PET/MRI demonstrated a combined sensitivity of 0.67 (95% CI:0.50-0.82), while mpMRI had a sensitivity of 0.51 (95% CI:0.40-0.62). This difference in sensitivity was also not significant (*P* = 0.12).

**Table 3:** Subgroup analysis based on ^68^Ga-PSMA PET/CT vs. mpMRI and ^68^Ga-PSMA PET/MRI vs. mpMRI in detecting local staging for prostate cancer.

Outcome	Imaging	Number of studies	Heterogeneity	Sensitivity(95%CI)	*P* Value between ^68^Ga-PSMA PET and mpMRI	Heterogeneity	Specificity(95%CI)	*P* Value between ^68^Ga-PSMA PET and mpMRI
ECE	^68^Ga-PSMA PET/CT vs. mpMRI				0.17			0.78
	^68^Ga-PSMA PET/CT	4	I^2^ = 47%	0.43 (0.24,0.64)		I^2^ = 81%	0.78(0.56,0.95)	
	mpMRI	4	I^2^ = 62%	0.66 (0.41,0.87)		I^2^ = 0%	0.75(0.66,0.83)	
	^68^Ga-PSMA PET/MRI vs. mpMRI				0.12			0.41
	^68^Ga-PSMA PET/MRI	4	I^2^ = 60%	0.67(0.50,0.82)		I^2^ = 0%	0.87(0.80,0.92)	
	mpMRI	4	I^2^ = 39%	0.51 (0.40,0.62)		I^2^ = 3%	0.90(0.84,0.95)	
SVI	^68^Ga-PSMA PET/CT vs. mpMRI				0.13			0.96
	^68^Ga-PSMA PET/CT	5	I^2^ = 54%	0.58 (0.37,0.77)		I^2^ = 76%	0.91(0.80,0.98)	
	mpMRI	5	I^2^ = 0%	0.76 (0.63,0.86)		I^2^ = 56%	0.91(0.83,0.97)	
	^68^Ga-PSMA PET/MRI vs. mpMRI				0.44			0.12
	^68^Ga-PSMA PET/MRI	3	I^2^ = 0%	0.68 (0.49,0.86)		I^2^ = 0%	0.93(0.88,0.97)	
	mpMRI	3	I^2^ = 0%	0.57 (0.37,0.76)		I^2^=10%	0.97(0.93,0.99)	

ECE, extracapsular extension; SVI, seminal vesicle infiltration.

It is demonstrated that ^68^Ga-PSMA PET/CT had a combined specificity of 0.78 (95% CI:0.56-0.95) for assessing ECE specificity, while mpMRI had a specificity of 0.75 (95% CI:0.66-0.83). The specificity difference between these techniques was not statistically significant (*P* = 0.78). Additionally, ^68^Ga-PSMA PET/MRI exhibited a combined specificity of 0.87 (95% CI:0.80-0.92), compared to 0.90 (95% CI: 0.84-0.95) for mpMRI. This specificity difference was also not statistically significant (*P* = 0.41).

We found that ^68^Ga-PSMA PET/CT had a sensitivity of 0.58 (95% CI:0.37-0.77) for detecting SVI in prostate cancer, while mpMRI had a sensitivity of 0.76 (95% CI:0.63-0.86). The difference in sensitivity was not considered statistically significant (*P* = 0.13). Additionally, the sensitivity of ^68^Ga-PSMA PET/MRI was 0.68 (95% CI:0.49-0.86) compared to mpMRI’s 0.57 (95% CI:0.37-0.76), with no significant difference observed (*P* = 0.44).

Our analysis of SVI specificity revealed that ^68^Ga-PSMA PET/CT and mpMRI both had a specificity of 0.91 (95% CI:0.80-0.98 and 0.83-0.97, respectively). The difference in specificity between these imaging techniques was not significant (*P* = 0.96). Additionally, ^68^Ga-PSMA PET/MRI had a specificity of 0.93 (95% CI: 0.88-0.97) while mpMRI had a specificity of 0.97 (95% CI:0.93-0.99), with no statistically significant difference in specificity (*P* = 0.12). All data are presented in **Table 3**.

### 3.8 Publication bias in ^68^Ga-PSMA PET and mpMRI in the identification of extracapsular extension and seminal vesicle invasion in cases of prostate cancer

The analysis of funnel plot asymmetry showed that there was no significant publication bias detected for the majority of outcomes, as all Egger’s test findings were above 0.05.Nevertheless, there were signs of bias in the publication of data regarding the sensitivity of ^68^Ga-PSMA PET in identifying ECE and SVI, as shown by Egger’s test results of below 0.001 and 0.05, respectively (refer to **Supplementary Figures 7-14**).

## 4 Discussion

In the context of early prostate cancer diagnosis according to the NCCN guidelines, the importance of using multiparametric MRI (mpMRI) in clinical decision-making before biopsy is emphasized (30). However, there’s conflicting evidence in the literature exists regarding the accuracy of mpMRI versus ^68^Ga-PSMA PET/CT in identifying extraprostatic extension. Yilmaz et al. (24) found that mpMRI is more accurate than ^68^Ga-PSMA PET/CT in detecting extraprostatic extension, with a detection rate of 87.5% compared to 66.7%. Conversely, Chen et al. (25)’s study suggests a different outcome. mpMRI showed a lower sensitivity compared to ^68^Ga-PSMA PET/CT for ECE, with rates of 54% and 78% respectively. These contentious conclusions have sparked our research interest in this subject.

In our meta-analysis examining the diagnostic performance of mpMRI and ^68^Ga-PSMA PET in detecting ECE and SVI in primary prostate cancer, we found comparable efficacy between these imaging modalities. In 2023, a comparison study was carried out by Wang et al. (31) on PSMA PET/CT and mpMRI in patients with localized prostate cancer, with reported results consistent with our own findings. Additionally, Kalapara et al. (32) and Ren et al. (33) also supports these conclusions. It is shown that there is no notable distinction between the two diagnostic methods in identifying or pinpointing primary prostate tumors.

Interestingly, in the study by Chow et al. (34) the findings somewhat diverge from ours: PET/MRI demonstrated higher sensitivity in detecting ECE and SVI—78.7% for PET/MRI versus 52.9% for mpMRI in ECE detection, and 66.7% for PET/MRI versus 51.0% for mpMRI in SVI detection—yet exhibited slightly lower specificity than mpMRI (82.2% for PET/MRI versus 86.2% for mpMRI in ECE specificity). Compared to mpMRI, PET/CT seems to possess lower sensitivity in detecting ECE and SVI, with PET/CT at 51.5% versus 61.0% for mpMRI in ECE detection, and 44.9% for PET/CT versus 61.8% for mpMRI in SVI detection. The discrepancy in conclusions between our study and Chow et al.’ research may stem from the specific delineation of ^68^Ga-PSMA as the radiotracer in our study, whereas Chow’s paper defined PSMA as the contrast agent, leading to differences in the inclusion criteria. Additionally, due to the variation in search timelines, our paper incorporated the most recent research findings. Variations in patient age, risk profiles, imaging protocols, and interpretation criteria across studies, as well as the time interval between different imaging modalities, are also significant (35).

In our head-to-head meta-analysis, several strengths distinguish our study from previous researches. The primary benefit is the ability to perform a comprehensive and trustworthy direct comparison between ^68^Ga-PSMA PET and mpMRI. This approach minimizes biases associated with non-comparative studies. Our study effectively eliminates potential confounding effects by specifically concentrating on ^68^Ga-PSMA as the radiotracer, rather than using other tracers. Thirdly, we further subdivided the PET analysis into two subgroups: PET/CT and PET/MRI. By comparing each subgroup separately with mpMRI, we found no statistically significant differences in the diagnostic performance of both types of PET modalities compared to mpMRI. The subgroup analysis conducted in our study further refines the diagnostic performance of ^68^Ga-PSMA PET, thereby enhancing the interpretability of results and increasing the applicability of our conclusions.

In addition, the two different diagnostic tools each have their own advantages and disadvantages. ^68^Ga-PSMA PET is recognized for its high sensitivity in detecting prostate cancer cells, making it particularly useful in identifying metastatic or recurrent disease, while mpMRI excels in detailed soft-tissue characterization, which is crucial for local staging and guiding biopsy (12). However, both methods have limitations. The accuracy of mpMRI for diagnosis can be influenced by the subjective nature of the PI-RADS scoring system, which may lead to inter-observer variability (36). In contrast, ^68^Ga-PSMA PET can effectively distinguish ISUP grade groups using SUVmax values, although these values can vary between studies, potentially affecting consistency in clinical application (37). Practical factors like cost, availability, and accessibility also differ significantly. While ^68^Ga-PSMA PET is often pricier and less accessible than mpMRI, the latter may not always offer the necessary specificity in certain cases, particularly when evaluating extraprostatic extension (38). The findings suggest that a combined diagnostic approach using both ^68^Ga-PSMA PET and mpMRI may enhance accuracy and provide complementary information, leading to better decision-making in clinical practice (39).

Our thorough examination of ^68^Ga-PSMA PET and mpMRI in the setting of initial prostate cancer offers valuable insights, although it is important to recognize limitations due to the heterogeneity of studies, which could impact the relevance of our findings. Furthermore, 8 of the 10 included studies were retrospective, which may have introduced selection and recall biases and affected the reliability of the results. Additionally, our conclusions are based on a small sample size, with only 8 direct comparison studies for ECE and SVI, limiting the statistical power to detect significant differences. Therefore, more extensive and well-designed prospective studies are needed to confirm these findings and guide clinical implementation.

## 5 Conclusion

Our analysis of the data indicates that both mpMRI and ^68^Ga-PSMA PET exhibit comparable accuracy in detecting ECE and SVI in prostate cancer patients. Nevertheless, the limited study sample size calls for further, larger prospective studies to validate these findings.

## Data Availability

The original contributions presented in the study are included in the article/**Supplementary Material**. Further inquiries can be directed to the corresponding author.
