# Supplementary material for: Comparison of 68Ga-PSMA PET and mpMRI for prostate cancer local staging: a comprehensive review and direct meta-analysis
Source: Front Oncol. 2024 Nov 1;14:1410229. doi: 10.3389/fonc.2024.1410229 (PMC11563965; doi:10.3389/fonc.2024.1410229)

Supplementary Table 1 Search strategy in PubMed and Embase.

| Database | Search strategy |
| --- | --- |
| PubMed | ("Positron-Emission Tomography"[Mesh] OR “PET”[Title/Abstract] OR “positron emission tomography”[Title/Abstract]) AND ("Multiparametric Magnetic Resonance Imaging"[Mesh] OR “mpMRI”[Title/Abstract] OR “Multiparametric Magnetic Resonance Imaging” [Title/Abstract] OR “Multiparametric MRI”[Title/Abstract]) AND (“PSMA”[Title/Abstract] OR “Prostate specific membrane antigen”[Title/Abstract] OR “HBED-CC”[Title/Abstract]) AND ("Prostatic Neoplasms"[Mesh] OR “Prostatic Cancers”[Title/Abstract] OR “Prostatic Cancer”[Title/Abstract] OR “Prostate Cancers”[Title/Abstract] OR “Prostate Cancer” [Title/Abstract] OR “Prostatic Neoplasm”[Title/Abstract] OR “Prostate Neoplasm”[Title/Abstract] OR “Prostate Neoplasms”[Title/Abstract] OR “Prostate tumor”[Title/Abstract] OR “prostatic tumor”[Title/Abstract]) |
| Embase | ('positron emission tomography'/exp OR ‘pet’:ab,ti OR 'positron emission tomography':ab,ti) AND ('multiparametric magnetic resonance imaging'/exp OR ‘Multiparametric Magnetic Resonance Imaging’:ab,ti OR ‘mpMRI’:ab,ti OR ‘Multiparametric MRI’:ab,ti) AND ('prostate specific membrane antigen'/exp OR ‘psma’:ab,ti OR 'prostate specific membrane antigen':ab,ti OR 'hbed cc':ab,ti) AND ('prostate tumor'/exp OR 'prostatic cancers’:ab,ti OR 'prostatic cancer':ab,ti OR 'prostate cancers’:ab,ti OR 'prostate cancer’:ab,ti OR 'prostatic neoplasm’:ab,ti OR 'prostate neoplasm’:ab,ti OR 'prostate neoplasms’:ab,ti OR 'prostate tumor’:ab,ti OR 'prostate tumors’:ab,ti) |

Supplementary Figure1 Sensitivity Analysis of ^68^Ga-PSMA PET in Detecting ECE Sensitivity


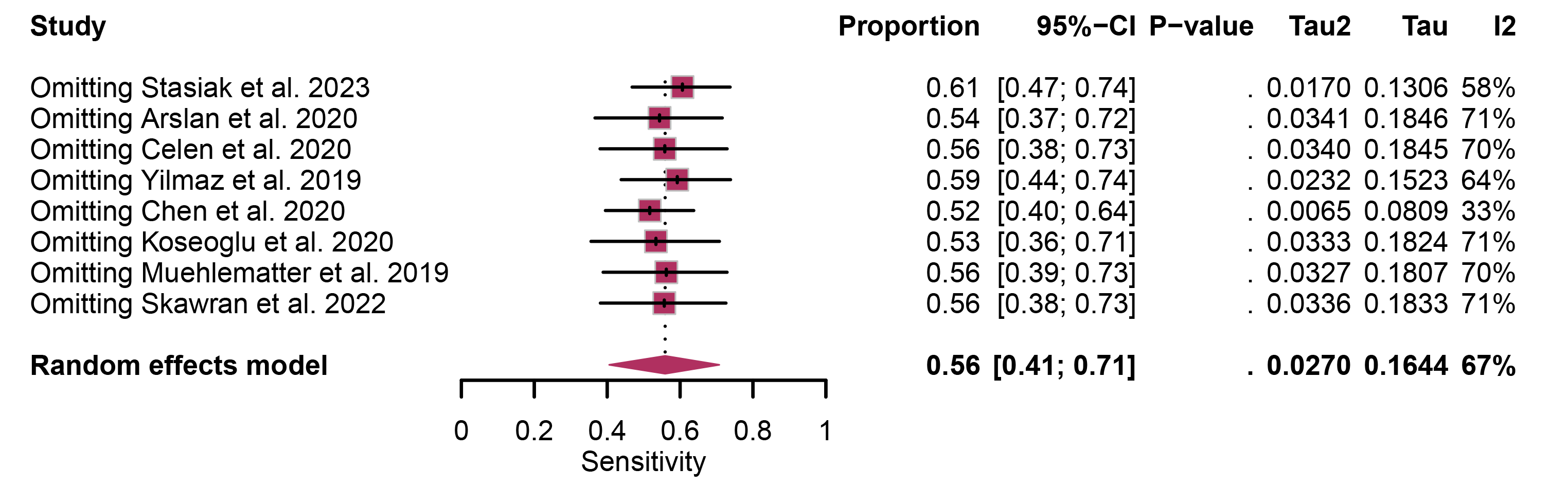


Supplementary Figure 2 Sensitivity Analysis of mpMRI in Detecting ECE Sensitivity


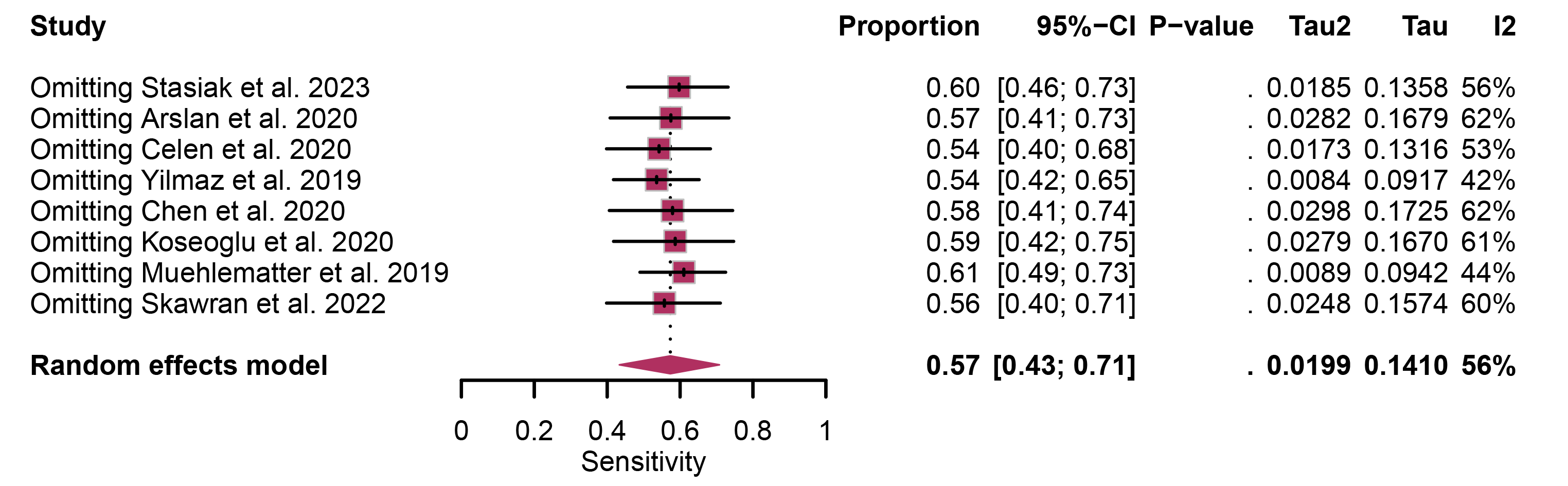


Supplementary Figure 3 Sensitivity Analysis of ^68^Ga-PSMA PET in Detecting ECE Specificity


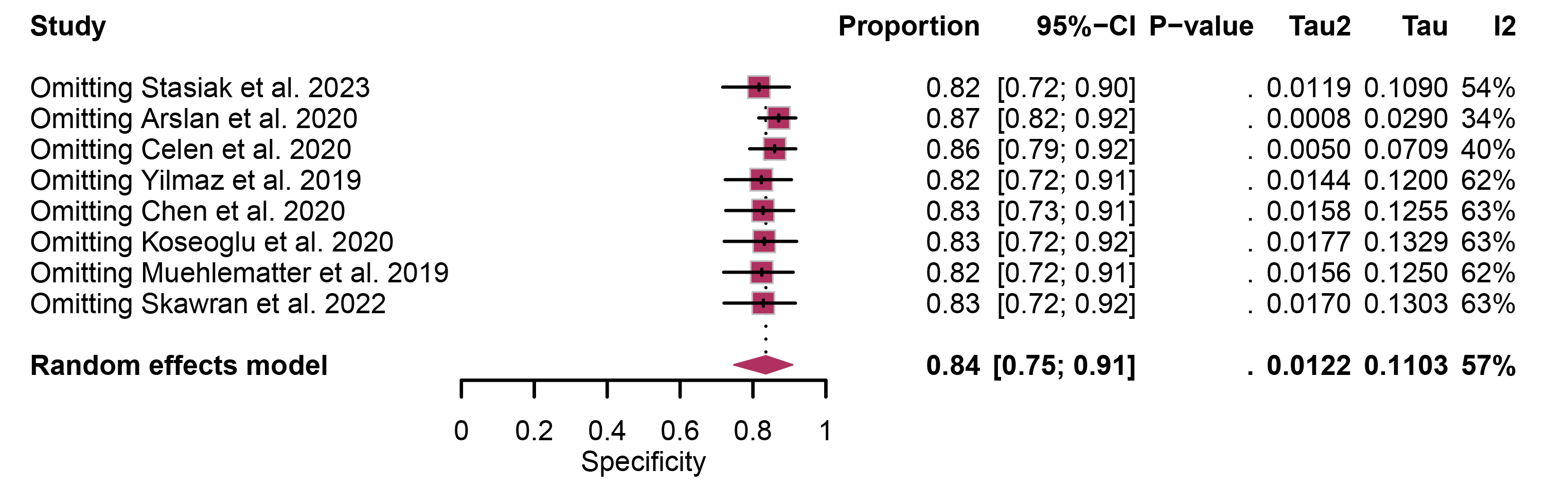


Supplementary Figure 4 Sensitivity Analysis of mpMRI in Detecting ECE Specificity


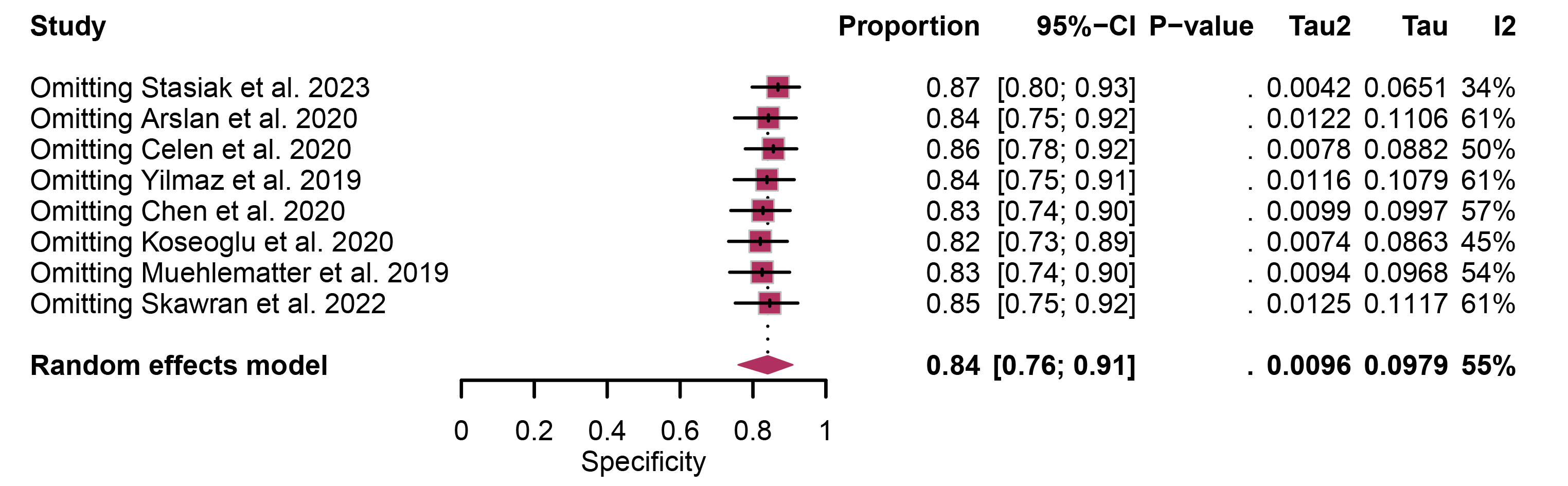


Supplementary Figure 5 Sensitivity Analysis of ^68^Ga-PSMA PET in Detecting SVI Specificity


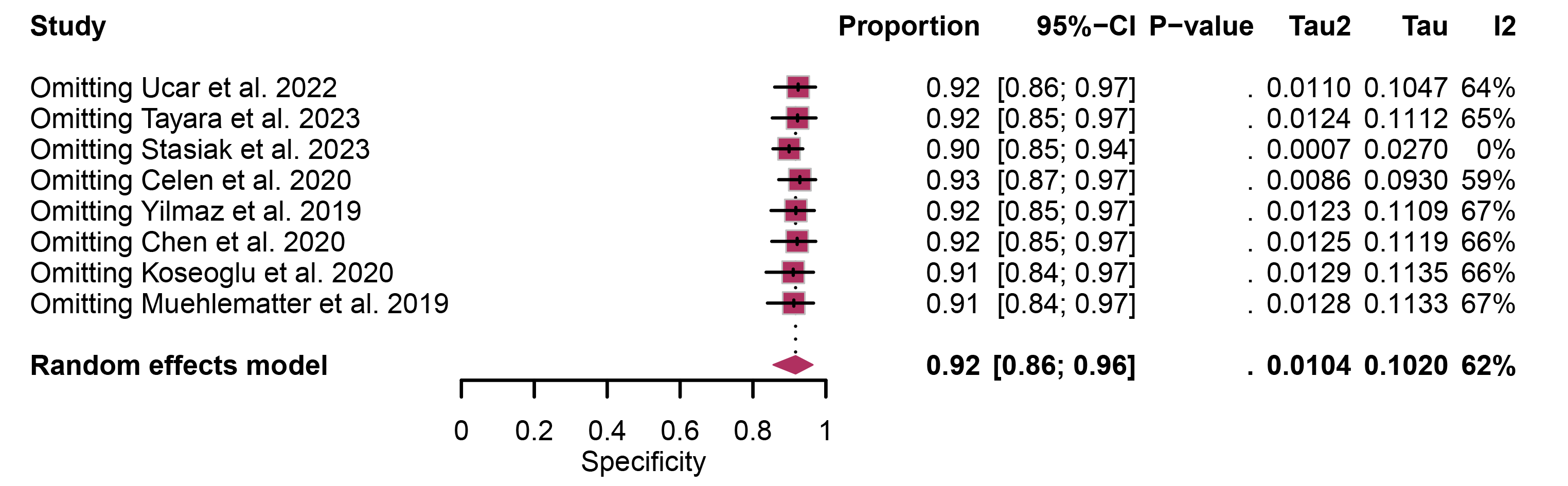


Supplementary Figure 6 Sensitivity Analysis of mpMRI in Detecting SVI Specificity


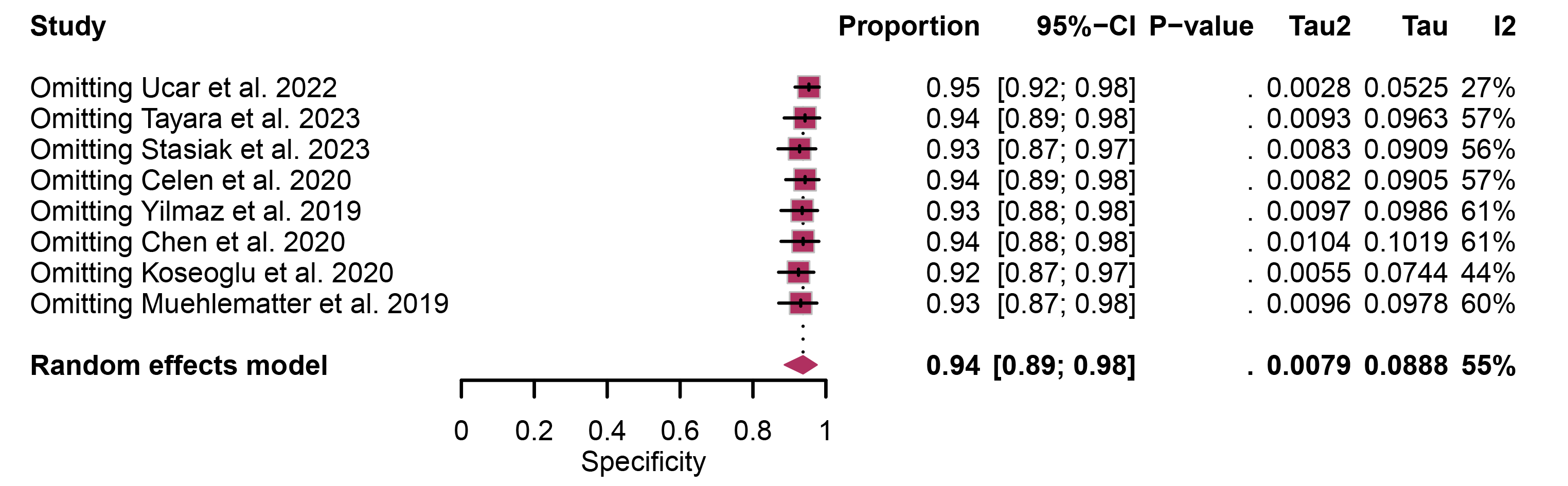


Supplementary Figure 7 Funnel Plot Demonstrating the Sensitivity of ^68^Ga-PSMA PET in Detecting Extracapsular Extension


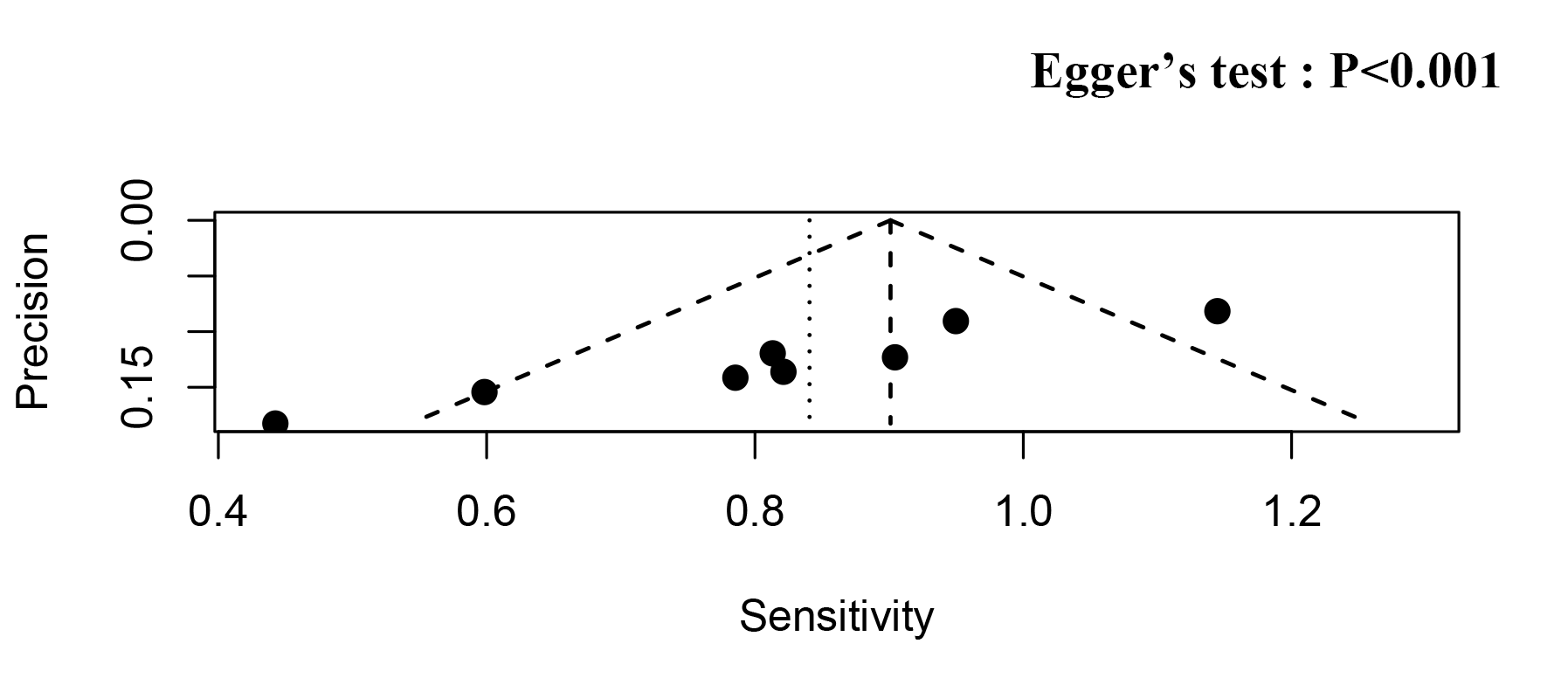


Supplementary Figure 8 Funnel Plot Demonstrating the Sensitivity of mpMRI in Detecting Extracapsular Extension


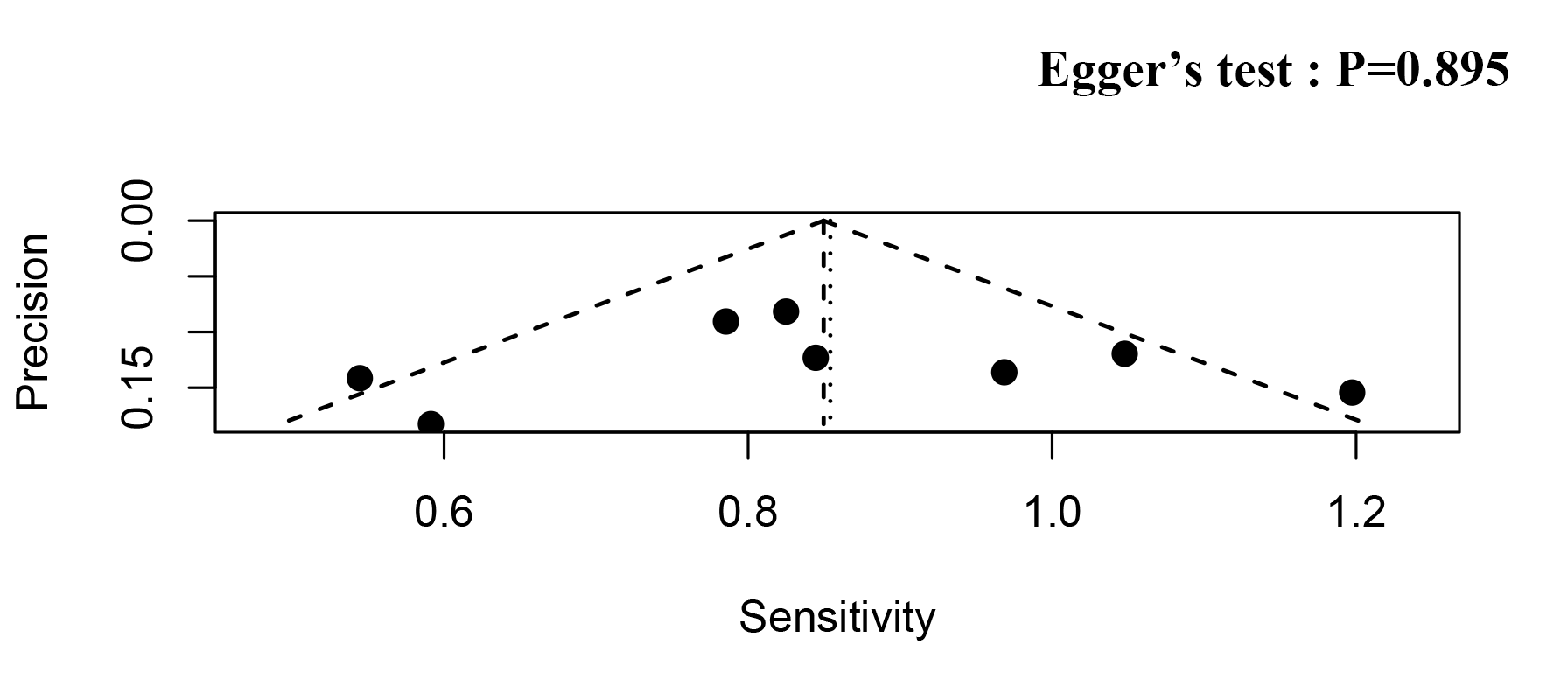


Supplementary Figure 9 Funnel Plot Demonstrating the Specificity of ^68^Ga-PSMA PET in Detecting Extracapsular Extension


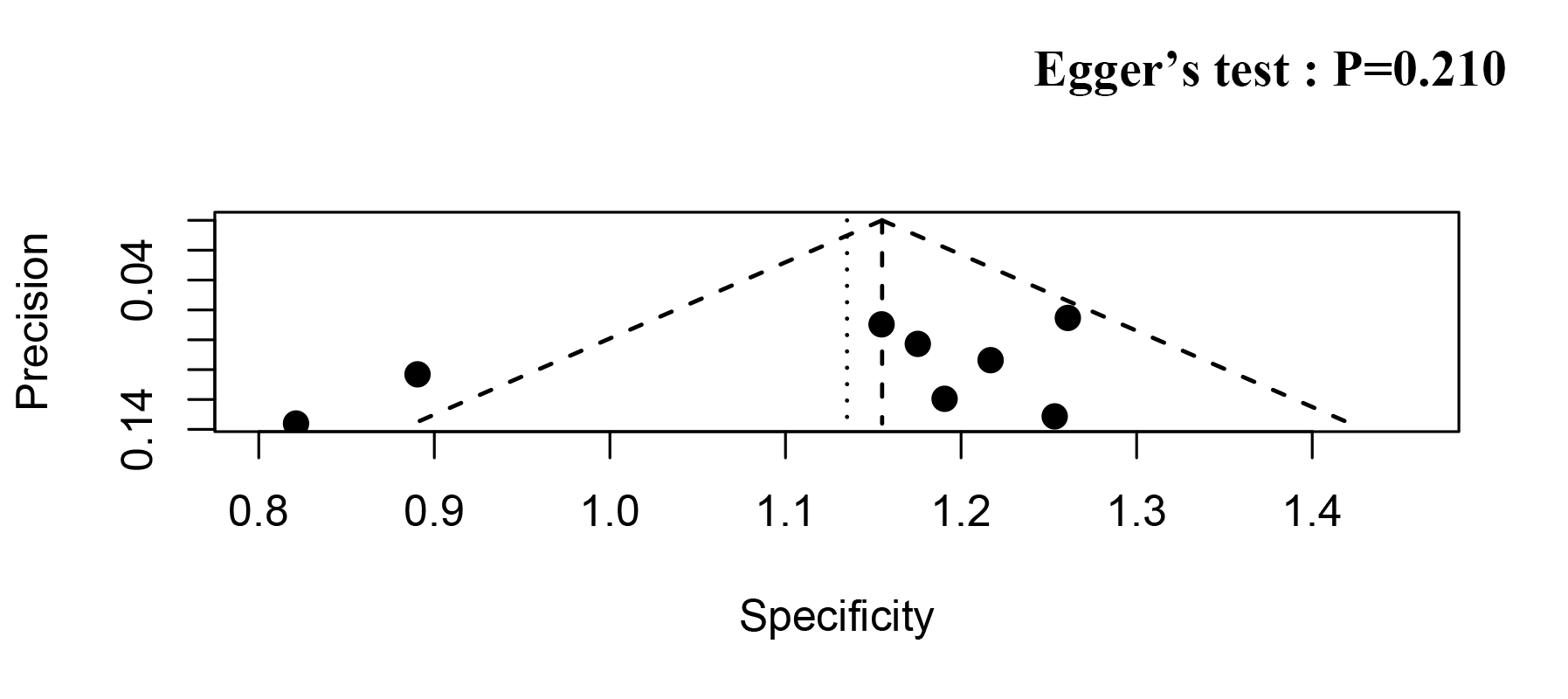


Supplementary Figure 10 Funnel Plot Demonstrating the Specificity of mpMRI in Detecting Extracapsular Extension


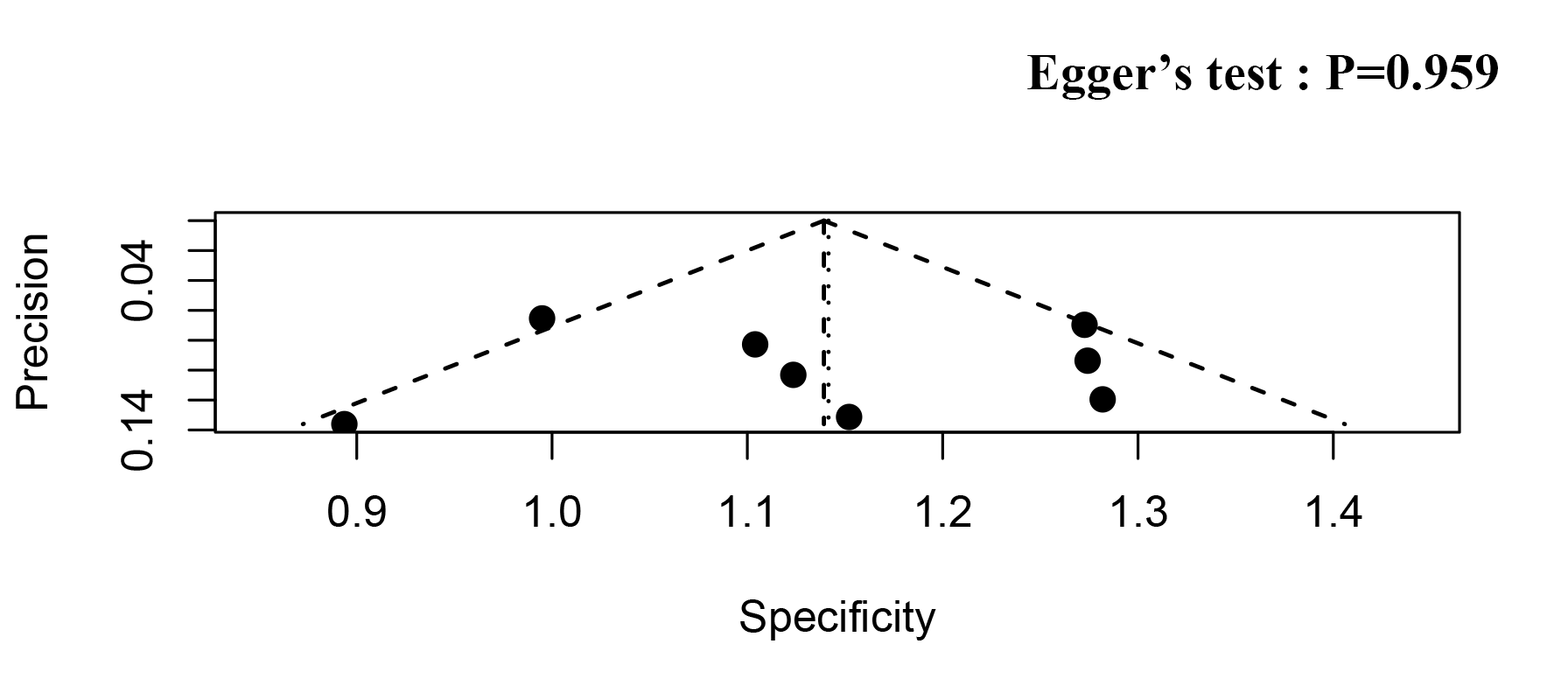


Supplementary Figure 11 Funnel Plot Demonstrating the Sensitivity of ^68^Ga-PSMA PET in Detecting Seminal Vesicle Invasion


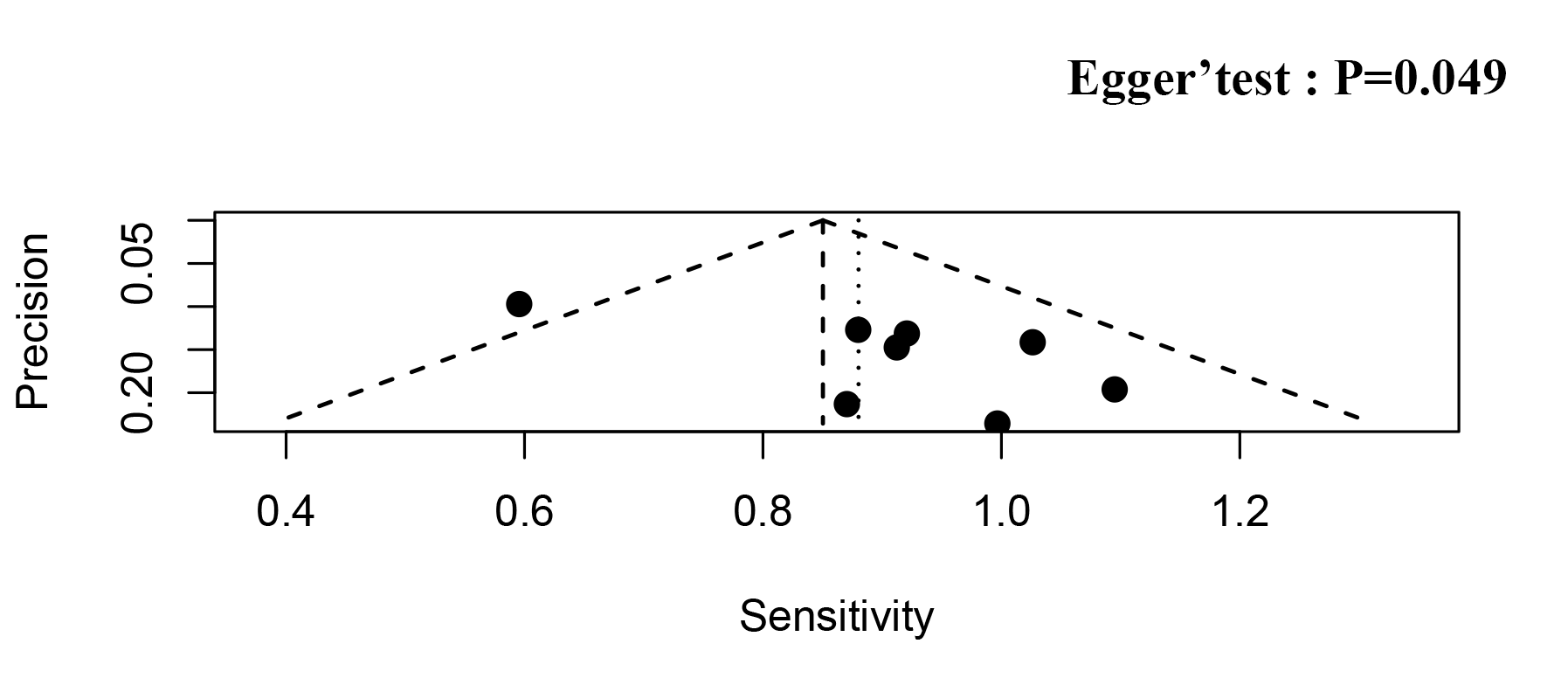


Supplementary Figure 12 Funnel Plot Demonstrating the Sensitivity of mpMRI in Detecting Seminal Vesicle Invasion


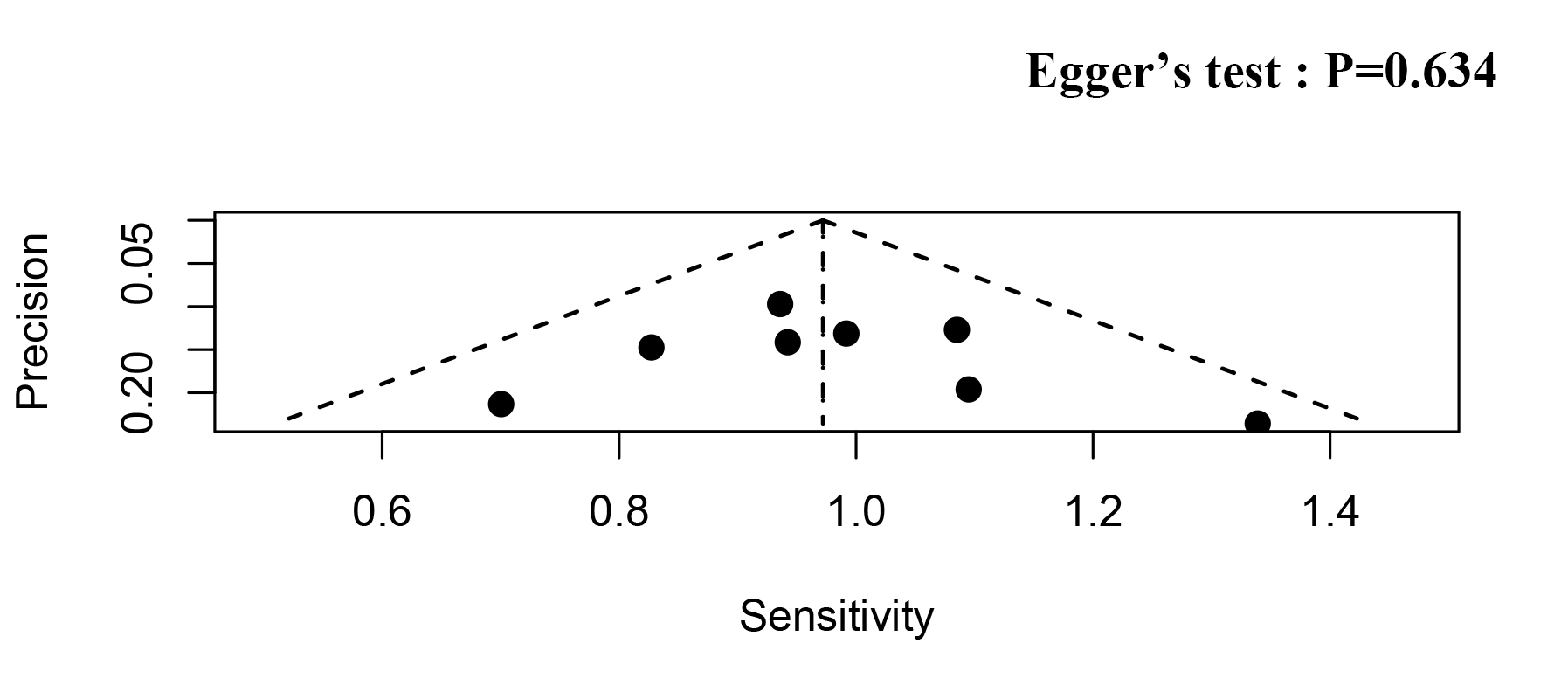


Supplementary Figure 13 Funnel Plot Demonstrating the Specificity of ^68^Ga-PSMA PET in Detecting Seminal Vesicle Invasion


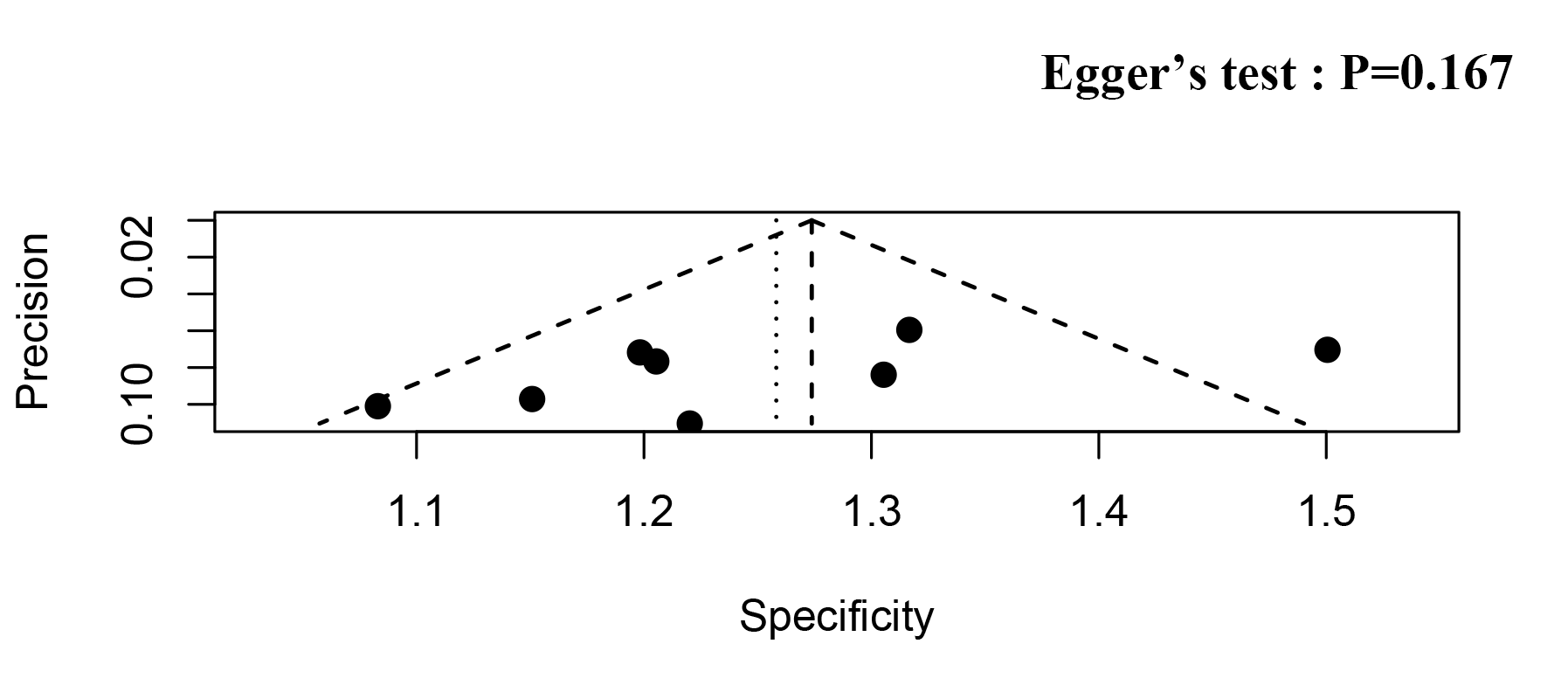


Supplementary Figure 14 Funnel Plot Demonstrating the Specificity of mpMRI in Detecting Seminal Vesicle Invasion


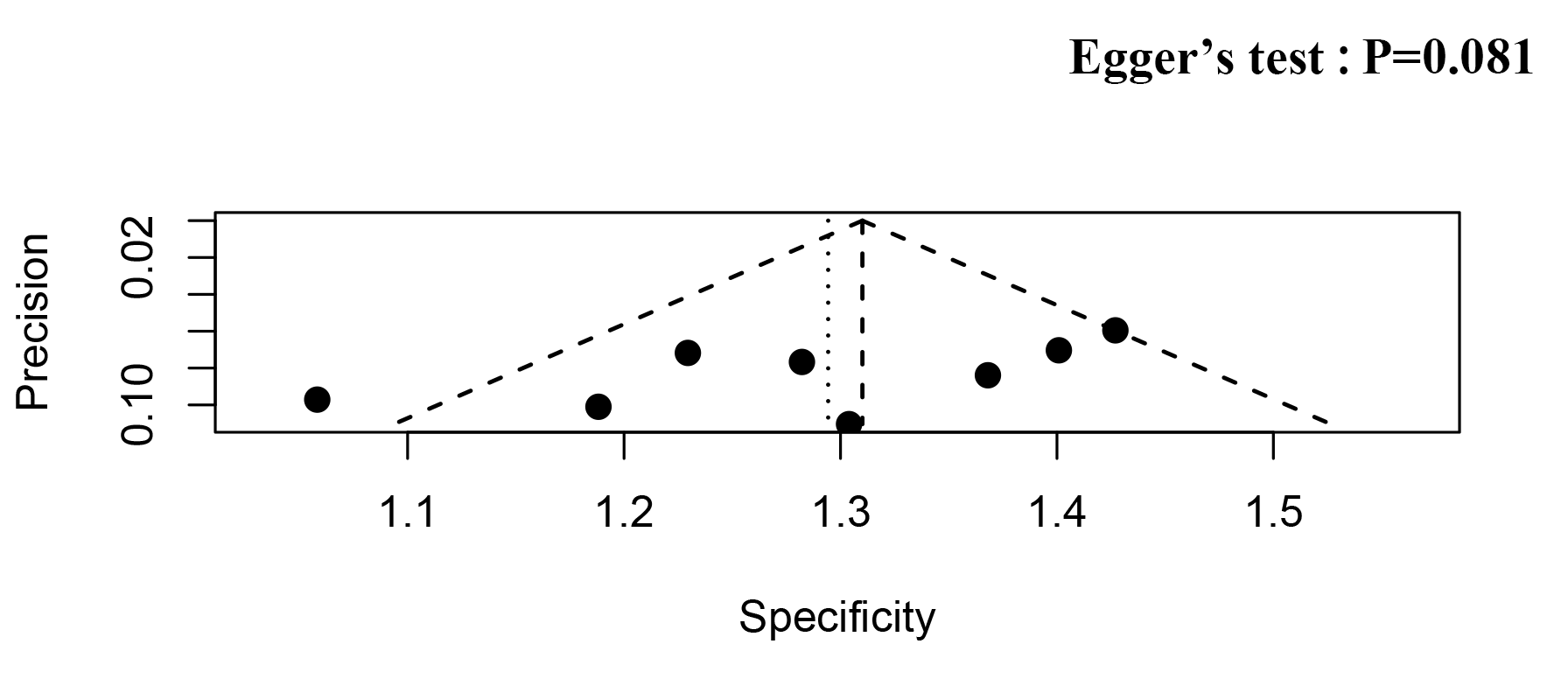

Supplement: Supplementary file 1 [file DataSheet1.docx]
